# Design of a pan-betacoronavirus vaccine candidate through a phylogenetically informed approach

**DOI:** 10.1126/sciadv.abq4149

**Published:** 2023-01-18

**Authors:** Eric Lewitus, Hongjun Bai, Morgane Rolland

**Affiliations:** ^1^ U.S. Military HIV Research Program, Walter Reed Army Institute of Research, Silver Spring, MD, USA.; ^2^ Henry M. Jackson Foundation for the Advancement of Military Medicine Inc., Bethesda, MD, USA.

## Abstract

Coronaviruses are a diverse family of viruses that crossed over into humans at least seven times, precipitating mild to catastrophic outcomes. The severe acute respiratory syndrome coronavirus 2 pandemic renewed efforts to identify strains with zoonotic potential and to develop pan-coronavirus vaccines. The analysis of 2181 coronavirus genomes (from 102 host species) confirmed the limited sequence conservation across genera (alpha-, beta-, delta-, and gammacoronavirus) and proteins. A phylogenetically informed pan-coronavirus vaccine was not feasible because of high genetic heterogeneity across genera. We focused on betacoronaviruses and identified nonhuman-infecting receptor binding domain (RBD) sequences that were more genetically similar to human coronaviruses than expected given their phylogenetic divergence. These human-like RBDs defined three phylogenetic clusters. A vaccine candidate based on a representative sequence for each cluster covers the diversity estimated to protect against existing and future human-infecting betacoronaviruses. Our findings emphasize the potential value of conceptualizing prophylaxis against zoonoses in terms of genetic, rather than species, diversity.

## Teaser

High genetic heterogeneity invalidates pan-coronavirus vaccine strategies but pan-betacoronavirus vaccines may be achievable.

## INTRODUCTION

In the past two decades, coronavirus (CoV) strains emerged from nonhuman hosts to infect humans three times with severe consequences: severe acute respiratory syndrome coronavirus (SARS-CoV) in 2003, Middle East respiratory syndrome coronavirus (MERS-CoV) in 2012, and SARS-CoV-2 in 2019. Another four CoVs, endemic in humans (alphaCoV 229E and NL63, betaCoV HKU1 and OC43), crossed over between the 13th and 20th century (*1*). These crossover events demonstrate that CoVs are diverse in the wild and can adapt to humans with deleterious outcomes.

CoVs are enveloped particles containing a positive-sense, single-stranded RNA of ~30 kb (*2*). They encode nonstructural proteins and four structural proteins: Spike (S), Envelope (E), Membrane (M), and Nucleocapsid (N). Four CoV genera diverged millions of years ago (*3*) and adapted to different animal hosts, with alphaCoVs and betaCoVs infecting mammals and gammaCoVs and deltaCoVs infecting both mammals and birds (*4*). While the error-prone RNA-dependent RNA polymerases of CoVs, similar to polymerases from other RNA viruses (*5*), fuel high substitution rates, this is inhibited by a CoV-unique replication proofreading mechanism, resulting in an overall lower substitution rate (*6*). Spillover between hosts has resulted in myriad recombination events in the natural history of CoVs, for example, between bats and pigs (*7*, *8*). Most recombinations were detected in S, which contains the receptor binding domain (RBD) (*9*). Recombination is documented in the evolution and possibly the emergence of MERS-CoV (*10*–*12*) and SARS-CoV (*13*–*15*). This may explain the differential receptor usage of CoVs: SARS-CoV and SARS-CoV-2, but not all sarbecoviruses (*16*, *17*), use angiotensin-converting enzyme 2 (ACE2); MERS-CoV uses dipeptidyl peptidase 4 (*18*, *19*); and HKU1 and OC43 both bind to sialoglycan-based receptors (*20*).

Concern for future viral crossovers incentivized many approaches for identifying potential zoonoses. The total estimated number of mammal-infecting viruses ranges widely, with 631,000 to 827,000 viruses with the potential ability to infect humans (https://ipbes.net/pandemics). These viruses, including zoonoses, are disproportionately identified in bats, rodents, and nonhuman primates, even when accounting for sampling biases (*21*, *22*). Zoonotic risk is often assigned on the basis of proximity of the host or similarity with known human-infecting viruses, although it is still unclear that viral host—or the ecological or phylogenetic context of a host—is a reliable proxy for zoonotic risk (*23*) and that the genome compositional similarity (e.g., frequency of CpG dinucleotides) of viruses to known human-infecting CoVs (hCoVs) can better identify viruses with crossover potential (*24*, *25*). Large-scale virological sampling efforts in the wild, which are becoming increasingly common and sophisticated (*26*, *27*), continue to identify previously unknown viruses, even among well-studied species (*28*). The identification of new viruses is not paralleled by an increase in virus families, suggesting that focusing on viral families, provided that the biological and known diversity within them is sufficient, holds clues to understanding zoonotic potential and provides a path to designing future vaccines.

The succession of SARS-CoV-2 variants emerging since the beginning of the pandemic is echoed in calls for pan-CoV vaccines. The rapid adaptation of SARS-CoV-2 to current vaccines with the emergence and ongoing adaptation of the resistant omicron variant since late 2021 highlight the need to better understand viral evolutionary pathways to ground the selection of sequences in future CoV vaccines on phylogenetically informed criteria. Here, we reconstructed the evolutionary dynamics of the four CoV genera across proteins. As the complex evolutionary trajectories of the four genera created an untractable problem for a pan-CoV design, we focused on betaCoV and used the inferred evolutionary dynamics of betaCoV S sequences to design a phylogenetically informed trivalent RBD betaCoV vaccine candidate.

## RESULTS

### Different evolutionary histories across CoV proteins

**Fig. 1.**
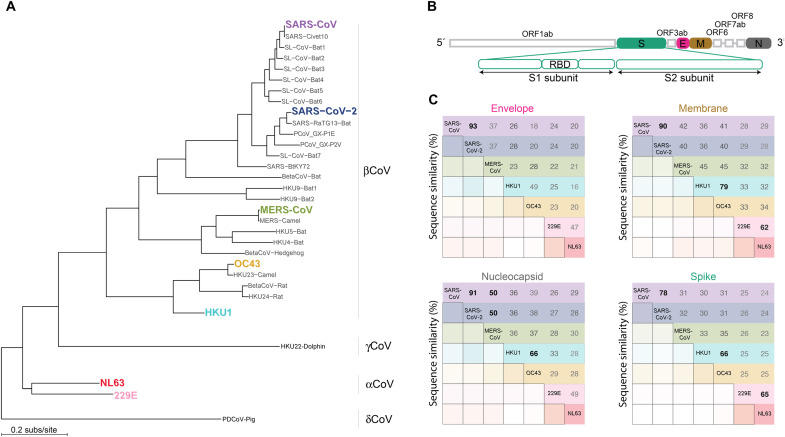
Low levels of sequence identity between hCoVs. (**A**) Phylogeny of seven hCoVs and 24 nonhuman-infecting CoVs recognized by the International Committee on Taxonomy of Viruses that span the four CoV genera. (**B**) Schematic of the SARS-CoV-2 genome, including the subunits of S. (**C**) Percentage of pairwise sequence identity of structural proteins for the reference sequences of seven hCoVs. Bottom is shaded by identity score (darker shades indicate higher scores); top enumerates the percentage sequence identity. Rows are colored according to hCoVs in (A); percentages of >50% are bolded.

**Fig. 2.**
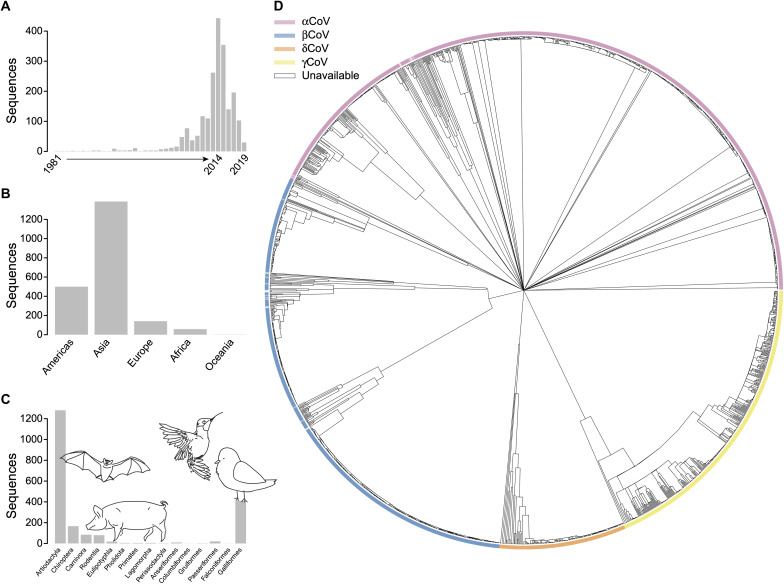
Whole-genome nucleotide sequences and phylogeny of global CoVs. Histograms of the (**A**) years, (**B**) landmasses, and (**C**) taxonomic orders from which whole-genome nucleotide sequences were sampled. Sketches of animals representing mammalian (bars 1 to 9) and avian (10 to 15) orders. (**D**) Phylogeny of 2181 whole-genome nucleotide CoV sequences, colored by genus (see legend).

We downloaded reference protein sequences for E, M, N, and S for the seven hCoVs. Overall amino acid sequence identity (pairwise gaps excluded) was low across hCoVs for E (23.9%), M (36.2%), N (36.3%), and S (29.5%) (Fig. 1). Among closely related hCoVs, sequence identity varied across proteins: It was highest for E (93%) and lowest for S (78%) between SARS-CoV and SARS-CoV-2, whereas it was highest for M (79%) but lowest for E (49%) between endemic hCoVs HKU1 and OC43. To further characterize CoV diversity, we analyzed 2181 whole-genome nucleotide sequences sampled between 1981 and 2019 predominantly from Asia (*n* = 1386) (Fig. 2, A to C). The samples included sequences from alphaCoV, betaCoV, gammaCoV, and deltaCoV (Table 1). The four genera emerged as monophyletic clades in the reconstructed phylogeny; 24 samples that had no associated genus metadata were placed in alphaCoV (*n* = 11), betaCoV (12), and gammaCoV (1) (Fig. 2D).

**Table 1.** Unique (<99% identity) number of sequences by genomic region and CoV genus.

Genera	Whole genome	Spike	Envelope	Membrane	Nucleocapsid
AlphaCoV	940	578	126	109	303
BetaCoV	651	132	47	81	129
GammaCoV	412	219	17	63	124
DeltaCoV	178	67	19	29	45

**Fig. 3.**
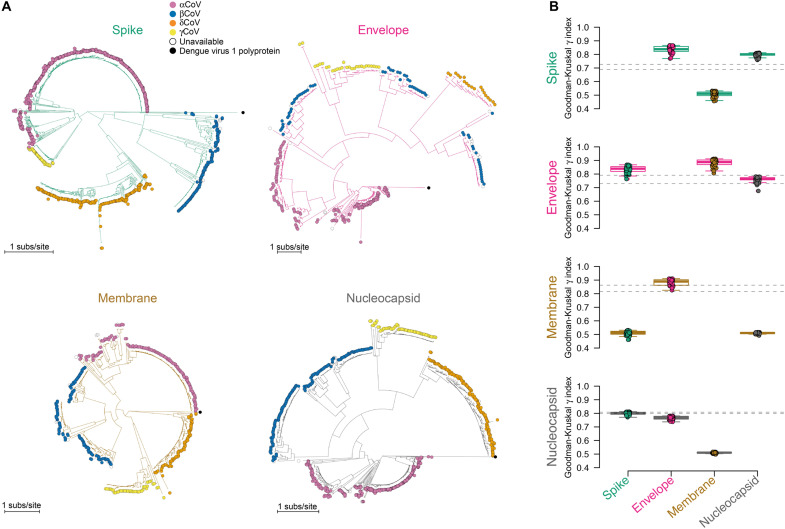
The evolutionary histories of CoV proteins S and M differ from a whole-genome reconstruction. (**A**) Maximum-likelihood phylogenies constructed for S, E, M, and N. Tips are colored according to genus (see legend). (**B**) Correlation coefficients based on the Goodman-Kruskal γ index for comparisons between phylogenies (for 50 permutation tests). Dashed lines indicate the maximum and minimum values for comparisons against the whole-genome phylogeny.

We analyzed phylogenies constructed from nucleotide and amino acid sequences to compare their diversification histories. Recombination breakpoints were identified for each genus in S: four breakpoints in alphaCoV [ΔAkaike information criterion (ΔAIC) = 9.89], four breakpoints in betaCoV (ΔAIC = 199.34), one breakpoint in deltaCoV (ΔAIC = 59.77), and three breakpoints in gammaCoV (ΔAIC = 76.03). A single-partition model was best supported across all iterations for E (median ΔAIC = 6488.97), M (median ΔAIC = 3551.34), and N (median ΔAIC = 2411.81). Phylogenies constructed with S, M, and N accurately clustered the four CoV genera in monophyletic clades, whereas betaCoVs were paraphyletic in the E phylogeny (Fig. 3A). Topological correlations between protein phylogenies (Fig. 3B), which ignore the branch-length differences expected for phylogenies constructed from proteins of different sizes, were computed with permutation tests against a null hypothesis that the phylogenies were identical (Goodman-Kruskal γ index = 1). Most comparisons showed no significant difference (*P* > 0.059), yet topological correlations were significantly different between M and S (*P* = 0.012) and M and N (*P* = 0.005), as well as between S and the whole-genome phylogeny (*P* = 0.018). This suggests rearrangements (due to protein-specific recombination or shifts in rates) in the deeper (ancient) evolutionary histories of M and S.

### Elevated substitution rate and accumulated diversity in the betaCoV S protein

**Fig. 4.**
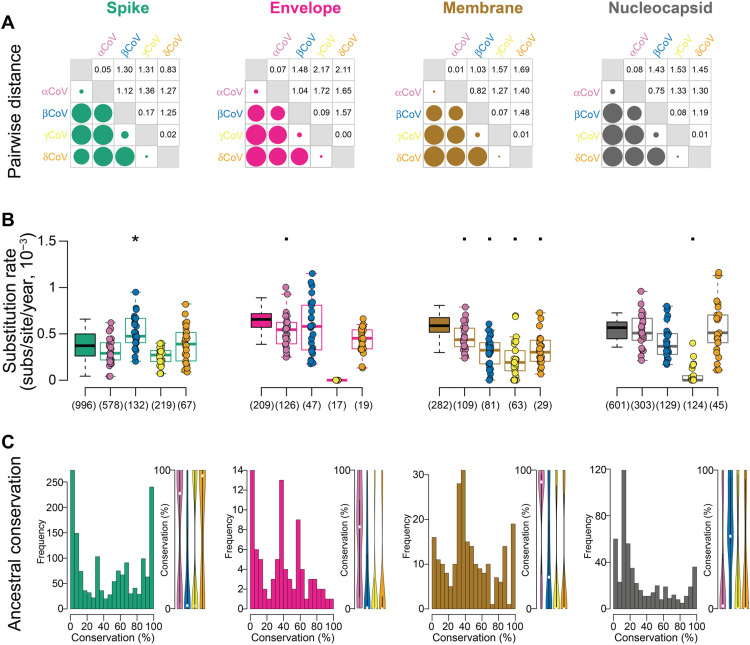
An elevated substitution rate in the betaCoV S protein has given rise to high accumulated diversity. (**A**) Median pairwise distances within each genus and between genera for each structural protein. Circles are scaled separately for each protein. (**B**) Boxplot of substitution rates (substitutions per site per year) with bootstrapped confidence intervals for each protein and genus. The solid box is calculated for all genera, and each jitter plot is calculated on a specific genus. Numbers below each box show the number of sequences analyzed. Asterisks and squares denote significantly higher and lower pairwise differences, respectively, between the all-genera and genus-specific rates (pairwise test, Bonferroni correction, *P* < 0.05). (**C**) Percentage of conservation across all sites for each protein. Histograms are calculated on all genera together, and violin plots are calculated on each genus separately; medians are figured with white circles.

Pairwise distances between protein sequences [calculated under the substitution model that showed the best fit, Jones-Taylor-Thornton (JTT)] showed similar patterns within and between genera across proteins (Fig. 4A). Median within-genera pairwise distances were low (≤0.17) for alphaCoV, gammaCoV, and deltaCoV across S, E, M, and N, whereas median within-genera pairwise distances for betaCoV were considerably higher across proteins (0.75 to 1.12). Mean between-genera pairwise distances were highest for E (1.78) and lowest for S (1.22), and ranking of between-genera distances for each genera was not uniform across proteins, consistent with differential selection on proteins in each genera, although the highest median between-genera distances were typically in alphaCoV (E, M, and N) and the lowest in deltaCoV (S, E, and N).

Substitution rates (substitutions per site per year) differed across proteins, yet differences in bootstrapped confidence intervals did not reach significance for protein-specific rates [analysis of variance (ANOVA), *P* = 0.056; Bonferroni correction, *P* > 0.058] (Fig. 4B). For each protein, there was significant genus-specific variability: For S, the rate for betaCoV strains was significantly higher than the rate inferred for all strains (Bonferroni-corrected *P* = 0.038); for E, M, and N, the rate for at least one genus was significantly lower than the rate inferred for all strains (*P* < 0.033).

Sites conserved with the most recent common ancestor (MRCA) were distributed differently between proteins (Kolmogorov-Smirnov test, *P* < 0.0003), except between E and M (*P* = 0.124), with the highest median conservation in S (50.6%) and the lowest in N (19.7%) (Fig. 4C). For S, the median conservation was significantly lower (Mann-Whitney *U* test, *P* < 0.001) in betaCoV (2.4%) and gammaCoV (2.2%) than in alphaCoV (83.3%) and deltaCoV (96.1%). AlphaCoV conservation was significantly higher than other genera (*P* < 0.001) for E (59.2%) and M (91.6%), whereas betaCoV conservation was higher (*P* < 0.001) than other genera for N (52.4%). The lower conservation seen in betaCoV and gammaCoV S was not uniform. The median MRCA conservation was significantly lower (*P* < 0.001) for S1 than S2 in alphaCoV (median S1 = 7.9%, S2 = 91.4%) and in betaCoV (S1 = 28.2%, S2 = 53.4%) but not meaningfully different in gammaCoV (S1 = 1.7%, S2 = 3.5%) or deltaCoV (S1 = 96.1%, S2 = 98.7%) (fig. S1). Ancestral conservation of S1 and RBD (28.1%) was similar (*P* = 0.057) for betaCoV. Thus, the evolutionary dynamics of S are not universal to CoV genera.

### Large yet representative sampled diversity across hCoVs

To determine whether our protein alignments reflected actual diversity in the wild, we developed a method for estimating missing site-specific diversity in protein alignments. We tested this method on simulated alignments of 5000 to 50,000 sequences, which were downsampled by 9 to 99%. Mean of median values for estimated diversity, *D*_e_, across all alignment sizes deviated by <1, and typically by zero, from actual diversity for all simulated alignments with sampling fractions of ≥2% (fig. S2, A and B). Sampling fraction and the number of simulated sequences had similar effects on accurate recovery (Student’s *t* test, *P* = 0.92), although the effect of sampling fraction was more pronounced when there were fewer simulated sequences (fig. S3). Similarly, despite considerably different rarefaction rates for alignments simulated under different transition rates (fig. S4), sampling fraction had a significantly larger effect (*P* = 0.04) on accurate recovery than transition rate (fig. S5).

Using this method, the sampled CoV sequences covered >90% of the total estimated site-specific diversity in E (92.9%), M (94.4%), and N (95.1%) (fig. S2, C to E). Coverage was high for S within each genus. For alphaCoV, 67 sites in S1 (including 17 in the RBD) and 14 sites in S2 had one inferred missing amino acid and four sites in S1 had two inferred missing amino acids, indicating that existing sequence data covered 93.4% of the total estimated diversity in S (fig. S2F). For betaCoV, 21 sites in S1 (including 5 in the RBD) had one inferred missing amino acid; hence, existing sequence data covered 98.4% of the total estimated diversity in S (fig. S2G). For gammaCoV, 18 sites in S1 and 10 sites in S2 had one inferred missing amino acid, and 1 site in S2 had two inferred amino acids; hence, existing sequence data covered 96.8% of the total estimated diversity in S (fig. S2H). For deltaCoV, no missing diversity was inferred (fig. S2I).

### BetaCoV RBD sequence similarity across large phylogenetic distances

**Fig. 5.**
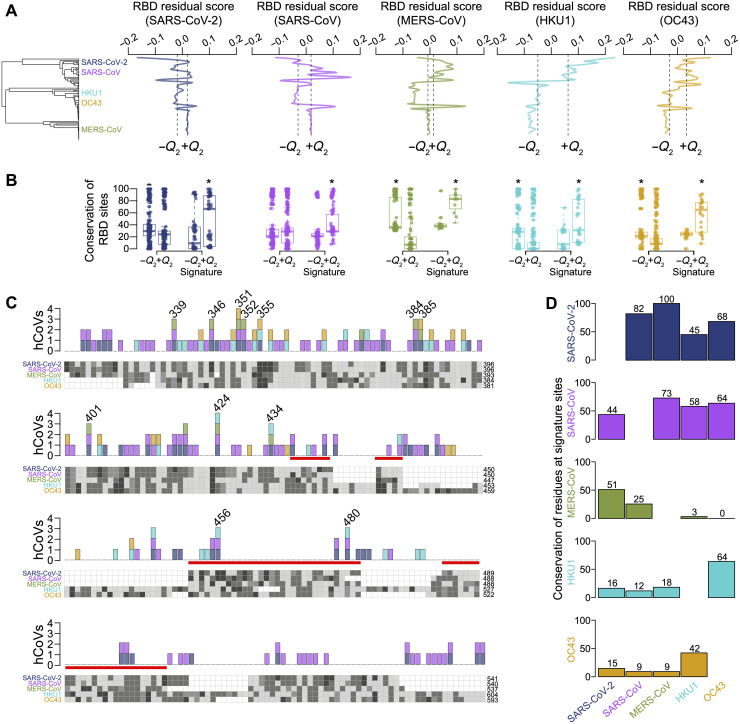
BetaCoV RBD sequence similarity across large phylogenetic distances. (**A**) Whole-genome phylogeny of betaCoVs with hCoVs labeled. Line plots of the distribution of RBD residual scores, ordered in line with the phylogeny as the *x* axis, for each hCoV, where higher scores indicate strains with an RBD closer to the hCoV than expected on the basis of phylogenetic distance. Dashed lines indicate the second quartile for residual scores of <0 (−*Q*_2_) and >0 (+*Q*_2_). (**B**) Percentage of conserved sites with each reference hCoV in (A) for −*Q*_2_ and +*Q*_2_ sequences for all RBD sites and only signature sites. Asterisks denote significantly higher values in pairwise comparisons (Mann-Whitney *U* test) between −*Q*_2_ and +*Q*_2_ sequences. (**C**) Graphical representation of aligned RBDs for hCoVs. The corresponding RBD site for each hCoV is shown at the end of each row. Each column is a site, and each cell represents the hCoV’s amino acid at that site with shades of gray corresponding to distinct residues (empty cells indicate gaps; the shades of gray do not consistently represent the same amino acid across sites but are used to differentiate them). A red bar covers the sites corresponding to the SARS-CoV-2 RBM. Stacked barplots (above) are colored according to (A) and show the number of hCoVs where the residue at the site is more conserved with respect to its target hCoV among +*Q*_2_ than −*Q*_2_ sequences (i.e., a signature site). Sites with more than two hCoVs represented are noted with the SARS-CoV-2 reference site. (**D**) Percentage of signature sites in each hCoV that have a matched residue in other hCoV.

We next simulated RBD evolution, beginning with the sequence for the betaCoV RBD MRCA, along a phylogeny constructed from S2 sequences, which is twice as conserved as S1 (fig. S6, A to F). The phylogeny of S2 sequences records the evolutionary divergence of S between hosts, whereas the phylogeny of RBD sequences records S host adaptation. We regressed the phylogenetic distance between each hCoV and simulated nonhuman strains on the simulated phylogeny against the distances between each hCoV and nonhuman strains on the phylogeny constructed from RBD sequences (fig. S6G). This produced residual scores for each nonhuman strain particular to each hCoV (fig. S6H). We took the negative of residual scores so that higher scores would indicate more similarity. Those with scores in the top 25th percentile of all positive scores (+*Q*_2_) were considered more similar to their target hCoV than expected, and those with scores less than the 75th percentile of all negative scores (−*Q*_2_) were considered less similar. SARS-CoV-2 had the most +*Q*_2_ sequences (*n* = 51), whereas MERS-CoV and HKU1 had the fewest (*n* = 29) (table S1); however, SARS-CoV-2 had the lowest median score for +*Q*_2_ sequences (0.019), whereas MERS-CoV had the highest (0.057) (Fig. 5A). For all hCoVs except SARS-CoV (*P* = 0.514), RBD sites were more conserved in −*Q*_2_ than +*Q*_2_ sequences (*P* < 0.020; Fig. 5B), emblematic of the close phylogenetic relatedness of many of these sequences to their target hCoV. However, for each hCoV, there was a subset of sites (+*Q*_2_ sites) in the RBD that were more conserved with respect to their target hCoV among +*Q*_2_ than −*Q*_2_ sequences (*P* < 0.001), ranging from 113 in SARS-CoV to 23 in MERS-CoV (table S1). For these sites, the mean of median differences between +*Q*_2_ and −*Q*_2_ sequences in conservation percentage was 27.2% across hCoVs. Of the 146 unique +*Q*_2_ sites, 37% were shared across more than one hCoV, including 12 that were shared across three hCoVs and 2 shared across four hCoVs (Fig. 5C). These signature sites together represent a constellation of genetic markers of zoonotic potential. Residue conservation at signature sites between hCoVs ranged widely (median = 42.8%), from 100% of SARS-CoV-2 signature sites matched by MERS-CoV to 0% of MERS-CoV sites matched by OC43 (Fig. 5D).

### Human-like RBDs primarily among sarbecoviruses

**Fig. 6.**
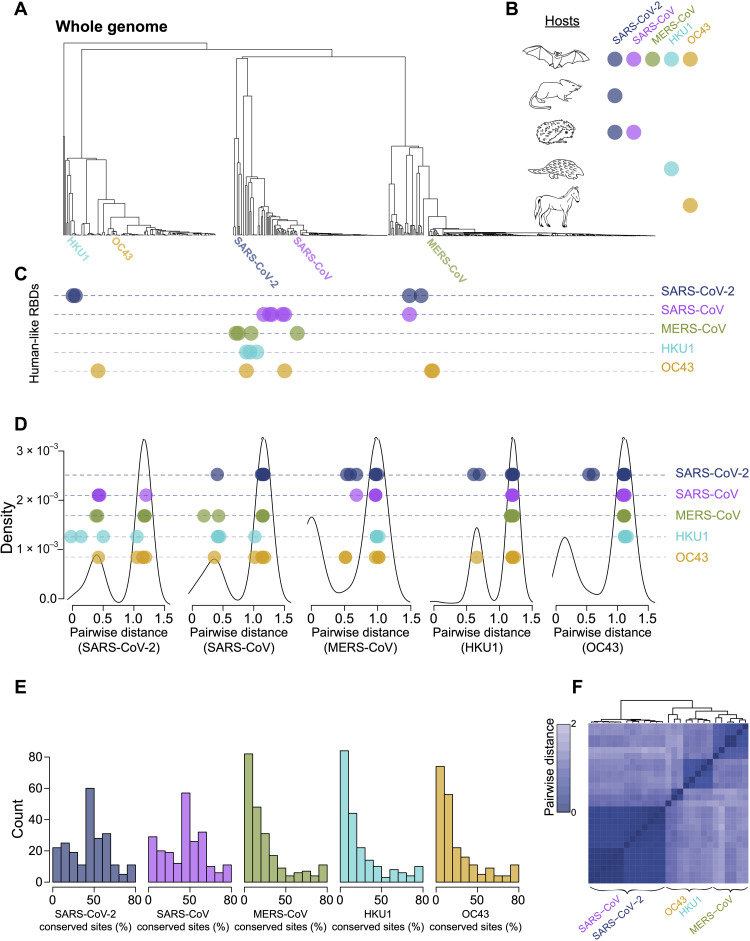
Human-like RBDs primarily among sarbecoviruses. (**A**) Whole-genome nucleotide betaCoV phylogeny with hCoVs labeled. (**B**) Sketches of host species; filled circles denote whether the human-like RBDs for each hCoV were sampled from that species. (**C**) Position in the phylogeny of the human-like RBDs for each hCoV. (**D**) Density plots of betaCoV RBD pairwise sequence distance for each hCoV. The pairwise distances of the human-like RBDs for each hCoV to each other hCoV is indicated by a filled circle. (**E**) Histograms of the percentage of consensus residues at each site in an RBD alignment of hCoVs and all human-like RBDs with respect to each hCoV. (**F**) Heatmap of the sequence distance matrix for an RBD alignment of hCoVs and human-like RBDs. Clusters containing each hCoV are noted.

For each hCoV, we identified nonhuman-infecting RBD sequences that were genetically more similar than expected on the basis of their phylogenetic divergence (so-called human-like RBDs) to those with the highest (top 5%) RBD residual scores, removing those with >97% identity (file S1). For SARS-CoV-2, HKU1, and OC43, human-like RBDs were phylogenetically distant, whereas those for SARS-CoV and MERS-CoV could be either phylogenetically distant or near (Fig. 6A). As expected, given that most sequences are isolated from bats, the human-like RBDs were predominantly derived not only from bats but also from mouse, hedgehog, pangolin, and horse (Fig. 6B), and the viruses were a mix of subgenera (Table 2). Nearly half of these most human-like RBDs (46.2%) were sarbecoviruses (Fig. 6C), as reflected by their relatively low median distance to SARS-CoV-2 (Mann-Whitney *U* test, *P* = 0.002) and SARS-CoV (*P* = 0.012) compared to all other RBD sequences (Fig. 6D). While a quarter (26.9%) of these most human-like RBDs were merbecoviruses, the median distance to MERS-CoV was not significantly less than the distance of MERS-CoV to other RBD sequences (*P* = 0.740) nor was that of HKU1 (*P* = 0.290) or OC43 (*P* = 0.972). Notably, the human-like RBDs for HKU1 included RaTG13 and PCoV_GX, which share 97.5 and 92.3% amino acid identity with SARS-CoV-2, respectively, and both SARS-CoV and SARS-CoV-2 identified a putative ancestor of MERS-CoV (coronavirus *Neoromicia*) among their human-like RBDs. When hCoVs were aligned with the human-like RBDs, the mean percentage of residues conserved with respect to each hCoV (Fig. 6E) was highest for SARS-CoV-2 (45.7%) and SARS-CoV (45.1%) compared to MERS-CoV (24.6%), HKU1 (24.2%), and OC43 (25.1%) (Mann-Whitney *U* test, *P* < 0.001), which was consistent with larger clustering around SARS-CoV-2 and SARS-CoV in the distance matrix of hCoVs and their human-like RBDs (Fig. 6F). This indicates that a putative RBD signature of human-infecting betaCoVs is most frequent in sarbecoviruses.

**Table 2.** The accession number, host species, and CoV subgenus for betaCoV human-like RBDs.

hCoV	Accession no.	Host species	CoV subgenus
SARS-CoV-2	YP_009513010.1	*Erinaceus europaeus*	Merbecovirus
	QQD78083.1	*Tylonycteris pachypus*	Merbecovirus
	AVP78031.1	*Rhinolophus pusillus*	Sarbecovirus
	ATQ39390.1	*Neoromicia capensis*	Merbecovirus
	AFO11507.1	*Mus musculus*	Embecovirus
	ACN89689.1	*Mus musculus*	Embecovirus
SARS-CoV	YP_009513010.1	*Erinaceus europaeus*	Merbecovirus
	ATO98120.1	*Rhinolophus sinicus*	Sarbecovirus
	AGC74176.1	*Chaerephon plicata*	Sarbecovirus
	ATO98181.1	*Rhinolophus sinicus*	Sarbecovirus
	ARO76382.1	*Rhinolophus hipposideros*	Sarbecovirus
	AGC74165.1	*Rhinolophus pusillus*	Sarbecovirus
MERS-CoV	YP_009273005.1	*Rousettus leschenaulti*	Nobecovirus
	ATQ39390.1	*Neoromicia capensis*	Merbecovirus
	ATO98132.1	*Rhinolophus sinicus*	Sarbecovirus
	QKF94914.1	*Eonycteris spelaea*	Nobecovirus
	BCG66627.1	*Rhinolophus cornutus*	Sarbecovirus
HKU1	YP_009072440.1	*Hipposideros pratti*	Hibecovirus
	QIA48623.1	*Manis javanica*	Sarbecovirus
	YP_003858584.1	*Rhinolophus blasii*	Sarbecovirus
	QHR63300.2	*Rhinolophus affinis*	Sarbecovirus
OC43	YP_009072440.1	*Hipposideros pratti*	Hibecovirus
	AUM60014.1	*Hypsugo savii*	Merbecovirus
	BAS18846.1	*Equus caballus*	Embecovirus
	ATO98181.1	*Rhinolophus sinicus*	Sarbecovirus
	AUM60024.1	*Pipistrellus kuhlii*	Merbecovirus

### A phylogenetically informed pan-betaCoV vaccine design

**Fig. 7.**
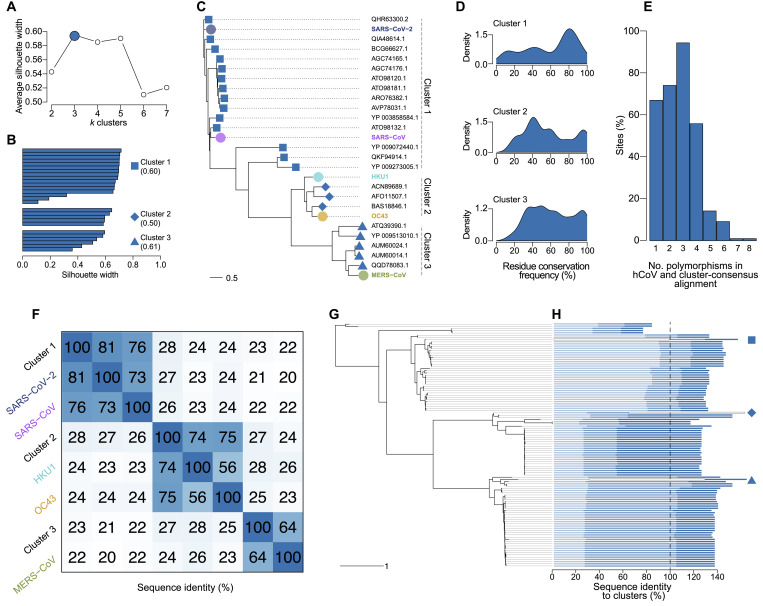
A phylogenetically informed pan-betaCoV vaccine design. (**A**) Average silhouette width of *k* clusters based on k-medoid clustering of the human-like RBD distance matrix. The optimal number of clusters, as determined by the highest average silhouette width, is marked in blue. (**B**) Silhouette widths for each human-like RBD sequence in its assigned cluster. (**C**) Maximum-likelihood phylogeny of human-like RBD sequences and hCoVs. The assigned cluster of each tip and the betaCoV hCoVs are indicated according to (B). (**D**) Distribution of the percentage of conserved residues at each site, excluding gaps, for each human-like RBD cluster alignment. (**E**) Percentage of sites with different numbers of polymorphisms in the alignment of human-like RBD cluster consensus sequences and betaCoV hCoV RBD sequences. (**F**) Sequence identity matrix for betaCoV hCoVs and the cluster consensus sequences. (**G**) Phylogeny for betaCoV RBD sequences and a corresponding (**H**) stacked barplot of sequence identity percentage between the consensus sequence for cluster 1 (light blue, square), cluster 2 (medium blue, diamond), and cluster 3 (dark blue, triangle) and all other sequences. The bars corresponding to each cluster are shown as empty. A dashed line is shown at 100% cumulative sequence identity for reference.

Because they are genetically similar to each hCoV RBD despite their phylogenetic divergence, the human-like RBDs identified above constitute key targets for a pan-betaCoV vaccine design. To select specific RBD sequences, we ran k-medoid cluster analysis on the distance matrix including all the human-like RBD sequences identified above and then computed consensus sequences for each cluster. These human-like RBDs were optimally clustered into three groups (Fig. 7A) with an average silhouette width of 0.57 (minimum = 0.50, maximum = 0.61) (Fig. 7B). SARS-CoV-2 and SARS-CoV were nested in the first cluster, HKU1 and OC43 in the second, and MERS-CoV in the third (Fig. 7C). The median percentage of conservation of residues across nongapped sites was highest in the first cluster (78.6%), followed by the second (67%) and third (60%) clusters, indicative of greater diversity in the second and third clusters (Fig. 7D), despite the first cluster being larger than the other two combined. We then computed majority consensus sequences for each cluster, removing sites with >80% gaps and drawing from a multinomial probability distribution of residues at sites with no majority residue (file S2). The number of sites with >80% gaps was 65 of 223, 78 of 304, and 9 of 223 in the first, second, and third cluster, respectively, and the number of ambiguous sites was 0, 10, and 5. When the cluster consensus sequences and hCoV RBD sequences were aligned, 78.9% of nongapped sites were polymorphic, but only one site was polymorphic in seven of eight sequences (and none in all sequences) (Fig. 7E). The median sequence identity across hCoVs and cluster consensus sequences was 24.9% (minimum = 17.4%, maximum = 73.4%) (Fig. 7F). The mean of median distances of hCoVs to cluster sequences was 25.1% (minimum = 24.1%, maximum = 28.6%), but within clusters, distances were typically higher: SARS-CoV-2 and SARS-CoV were similarly distant to their closest cluster consensus (81.1 and 75.7%, respectively); similar patterns were seen for HKU1 and OC43 (73.7 and 75.3%), while MERS-CoV shared the lowest identity with its closest cluster-consensus sequence (63.8%). Last, we aligned the cluster consensus sequences to the betaCoV RBD sequences and constructed a phylogeny (Fig. 7G). We computed the sequence identity between each cluster consensus sequence and all other sequences (Fig. 7H). As expected, the median sequence identity for each was quite low (23.1 to 25.3%), but the average cumulative sequence identity across sites was 84.5%, indicating that, together, the cluster consensus sequences largely cover betaCoV RBD diversity. To put this design in perspective with a current multivalent vaccine strategy, we reconstructed a phylogeny of RBD betaCoV sequences that included the five human betaCoV, our cluster consensus sequences, and the eight sequences present in the Mosaic-8b vaccine candidate developed at Caltech (*29*). The median pairwise distance between Mosaic-8b sequences represented about one-fifth of the distance between cluster consensus sequences (and 18.4% of the median distance between all betaCoV strains) (fig. S7)—the limited coverage of betaCoV diversity in Mosaic-8b is expected because the Mosaic-8b candidate is a pan-sarbecovirus vaccine candidate.

**Fig. 8.**
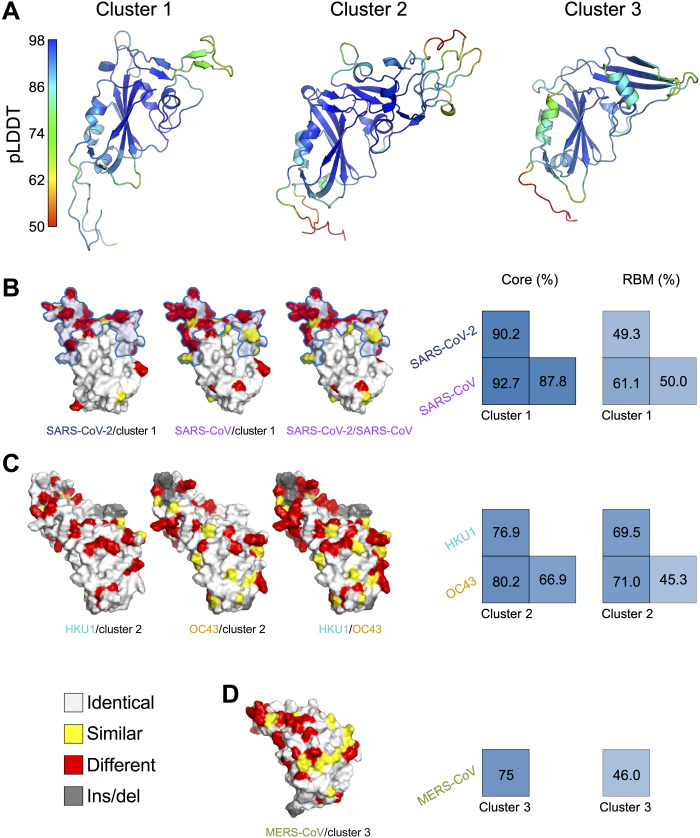
Structural resolution of derived pan-betaCoV vaccine sequences. (**A**) Predicted structure of cluster consensus sequences, colored by pLDDTs. Sequence differences between (**B**) cluster 1, SARS-CoV, and SARS-CoV-2; (**C**) cluster 2, HKU1, and OC43; and (**D**) cluster 3 and MERS-CoV mapped onto the predicted structures; and associated correlation plots for the core RBD and RBM. In the predicted structures, the RBM is indicated by a blue outline for cluster 1, SARS-CoV-2, and SARS-CoV; sites that are identical (white), similar (yellow), or different (red), as well as insertions/deletions (gray), are shown.

Last, we characterized the structure of the derived cluster consensus sequences, referred to as cluster 1, cluster 2, and cluster 3. Resolving structures for the cluster consensus sequences was constrained by the paucity of nonhuman betaCoV strains with structural resolution in the Protein Data Bank (UniProt IDs: A0A1Z2WUW0, A0A6B9WHD3, A0A6G6A1M4, and X2JHN8) and relatively low maximum sequence identity (63.2 to 80.7%) between cluster consensus sequences and sequences that were manually curated by Swiss-Prot. The median predicted alignment error was acceptable (4.3 to 5.23), although each sequence had maxima around 30 in discrete regions (fig. S8). Predicted local distance difference tests (pLDDTs) showed generally good confidence across all domains (*30*), excluding the terminal ends, with median pLDDTs between 89.97 and 91.62: In cluster 1, two domains (SARS-CoV-2 sites 165 to 181 and 213 to 214) were modeled with low confidence (median pLDDT = 66.8); in cluster 2, two domains (203 to 205 and 220 to 222) were modeled with low confidence (59.41), and one domain (192 to 198) showed disorder (49.32); and in cluster 3, one domain (197 to 200) showed low confidence (67.4) (Fig. 8A). To evaluate the feasibility of designing antigens with pan-betaCoV coverage, we compared the sequence similarity between cluster consensus sequences and their closest hCoVs in the predicted structures. Cluster 1 shared a considerably lower identity with SARS-CoV and SARS-CoV-2 in the receptor binding motif (RBM) than in the core RBD; however, these identities were comparable to those between SARS-CoV and SARS-CoV-2 (Fig. 8B). Cluster 2 had comparable identity levels with both HKU1 and OC43 in the core and RBM, at a level higher than the identity between HKU1 and OC43 (Fig. 8C). Cluster 3 showed a higher core than RBM identity with MERS-CoV (Fig. 8D).

## DISCUSSION

We characterized the evolution of Coronaviridae to derive a sequence framework for designing a universal CoV vaccine. We showed that there is little global sequence similarity among hCoVs and different patterns across proteins, with a complex evolutionary history most apparent in S. Given this heterogeneity, we focused on betaCoV RBDs and developed an approach to identify the most human-like sequences among RBDs that maximizes phylogenetic divergence (i.e., human-like RBDs). The diversity found in these human-like RBDs defined three clusters, which allowed us to derive three cluster consensus sequences that can be used as candidate vaccines to summarize the diversity found in all betaCoV.

The first part of our study consisted in describing Coronaviridae evolution. Protein identity was very low across hCoVs with differences across proteins. This variation reflects not only a heterogeneity in the evolutionary histories of each protein, particularly among the earliest divergences of CoV genera, but also the more than twofold differences in between-genera diversity across proteins. Genus-specific trends in diversification were not consistent across proteins, which suggests that differential selection pressures acted on each genus. Substitution rates varied substantially across genera and proteins but typically remained below 1 × 10^−3^ substitutions per site per year; these values were below the rates for SARS-CoV-2 emerging variants of concern, which have been estimated to be between 0.46 × 10^−3^ and 8.47 × 10^−3^ substitutions per site per year (*31*, *32*), reflecting a regression to mean values over large evolutionary periods. In S, we observed different dynamics between S1 and S2 segments in each genus, with comparable frequencies of conserved residues in both segments for gammaCoV and deltaCoV but divergent frequencies in alphaCoV and betaCoV. The high substitution rate and bimodal frequency of conserved residues in betaCoV S revealed a complementarity between an adaptive S1, which contains the RBD, and a more neutrally evolving S2, suggesting that S1 may record the adaptive history to hosts, while S2 could record the evolutionary divergence between hosts of betaCoV S sequences. This dynamic between S1, particularly for the RBD, and S2 provided a framework for identifying adaptive genetic signatures of potential zoonosis among betaCoV RBD sequences, whereby we used the relatively neutrally evolving S2 phylogeny as a backdrop to identify the RBDs with crossover potential.

In the second part, we focused on RBD sequences from betaCoV and predicted RBDs with human-like features to be included in a vaccine designed to cover betaCoV diversity. Our focus on the RBD was motivated by the need to demonstrate our vaccine design approach on a relatively small segment (with palatable diversity) and its importance as a key vaccine target because of its role in viral entry and the predominance of RBD-specific antibodies among CoV-neutralizing antibodies. We evolved RBD-like sequences on the more neutrally evolving S2 phylogeny, beginning with the MRCA of betaCoV RBDs, to simulate how the betaCoV RBD would have evolved under curtailed selection pressures. We then compared the phylogenetic distance between the evolved sequences and the actual distance of sequences on the RBD phylogeny. We found 29 to 51 nonhuman-infecting betaCoVs that had RBD sequences more closely resembling a target hCoV than expected on the basis of this comparison. We referred to the top 5% of these for each hCoV as human-like RBDs. These human-like RBDs were dominated by sarbecoviruses. This approach recovered the two nearest neighbors of SARS-CoV-2, RaTG13 and PCoV_GX, which were identified through the HKU1-based search, as well as the putative ancestor of MERS-CoV (coronavirus *Neoromicia*), identified by SARS-CoV and SARS-CoV-2. This implies that there is a signature of human-infecting RBDs that is peppered throughout the betaCoV phylogeny. We posit that the sequences containing this human-like RBD signature can be used to design a vaccine against betaCoV strains, including both those currently circulating in humans and those with a high crossover potential.

To summarize the diversity across these human-like RBDs, we clustered the closest ones into three groups and derived the corresponding consensus sequence of each group, thus generating a pan-betaCoV trivalent RBD vaccine candidate. Each consensus shared between ~65 and 85% identity with at least one hCoV, and the alignment of hCoVs and consensus sequences had ~90% polymorphic sites. This provides a diverse but targeted set of sequences that contain a signature of zoonosis potential. Notably, one-third of the sites that were imputed to identify these human-like RBDs were shared across two or more hCoVs, demonstrating how essentialized zoonotic risk can be when put in the context of millions of years of evolution.

Our evolutionary reconstruction and the model derived from it are limited by the sequences available. These sequences are biased toward certain taxonomic groups and toward viral neighbors of human-infecting viruses. We estimated that, at each site, the sampled diversity was representative of the expected diversity in the wild, suggesting that our sample is sufficient for drawing meaningful conclusions about global CoV diversity. Nonetheless, there are doubtless many CoV strains that remain unsampled in the wild, and the SARS-CoV-2 pandemic has renewed efforts to catalog these. While viral surveillance efforts are important, they will not directly identify strains of importance as vaccine candidates. Assessing risk factors is subject to biases associated with the most widely studied viruses and relies on the opinions of experts studying those viruses (*33*), while scaling up efforts of sampling viruses endemic to nonhuman species is exceedingly costly and laborious (*34*). An explicit assumption of our model is to penalize phylogenetic relatedness at the expense of sequence similarity, which we justify by observing the divergent landscape of hCoVs and concluding that phylogenetic sister taxa of known hCoVs are not de facto superior candidates for zoonosis. Furthermore, our model does not account for human receptor usage. While SARS-CoV and SARS-CoV-2 use ACE2, we consider that this is not an essential condition of viral spillover as other human CoVs do not use ACE2 (*16*, *17*), and any vaccine designed for maximal coverage of betaCoVs should prevent infection irrespective of receptor usage. Another assumption of our work is that sequences from a given genetic space are needed in our design if coverage of that space is desired. Specifically, we have a genetic approach to identify sequences to optimize coverage, and immunological studies will be needed to characterize the overlap between the genetic and immunologic space. There is limited understanding of the immunologic space covered by CoV antigens as few studies have mapped responses using antigens beyond genetically close clade 1a and 1b sarbecoviruses (*35*). Our work did not address what combination of sequences could serve as a variant-proof SARS-CoV-2 vaccine. Because current variants show numerous escape mutations at critical antibody contact sites and escape most of the SARS-CoV-2–neutralizing antibodies (*36*, *37*), we imagine that future variants will likely show further escape from immune responses. Whether a variant-proof SARS-CoV-2 vaccine can be created with SARS-CoV-2 antigens that are resistant in the RBD (which is the most important target for neutralizing antibodies) remains to be demonstrated as the immunogenicity that can be induced by resistant antigens is poorly understood.

In summary, we developed an approach to optimize viral diversity coverage and predict genetic signatures of spillover potential, thereby providing a path to rational pan-CoV vaccine design. While there is a consensus on the urgent need for a “universal” CoV vaccine (*38*), what breadth of coverage such a vaccine would confer is less clear. The universal or pan-CoV vaccine label has been applied to multiple vaccine objectives, including (i) a SARS-CoV-2 vaccine adapted to new circulating variants, (ii) a variant-proof SARS-CoV-2 vaccine, (iii) a pan-sarbecovirus vaccine, (iv) a pan-betaCoV vaccine, and a (v) pan-CoV vaccine. The current SARS-CoV-2 vaccine approach is to adapt the initial vaccine (based on the ancestral Wuhan-Hu-1 strain) to match the currently circulating variant. This strategy is inherently reactive and retrospective, in contrast to the “pan-CoV vaccine” agenda, which focuses on protection against future threats (new variants or strains). Hence, the second objective is to create a variant-proof SARS-CoV-2 vaccine. Different approaches aim to yield a variant-proof vaccine: multivalent platforms or formulations with distinct variants (with or without the ancestral SARS-CoV-2 S), inserts designed to elicit cellular responses (such as N), or the ancestral SARS-CoV-2 S in a platform deemed to elicit broader and more potent immunity (*39*, *40*). It is important to note that these variant-proof strategies do not necessarily include distinct variant sequences because it is argued that a given vaccine candidate based on the ancestral SARS-CoV-2 antigen could offer superior immune responses that would cover a broad array of viruses. Hence, the RBD-sortase A-conjugated ferritin nanoparticle nanoparticle vaccine candidate developed at Duke University and based on the ancestral SARS-CoV-2 strain aims to be variant-proof (*40*). Other strategies seek to cover incrementally broader CoV space. A leading strategy seeking to confer pan-sarbecovirus coverage is the nanoparticle vaccine developed at Caltech (Mosaic-8b), which includes eight RBD sequences (*29*). This vaccine is based on sarbecovirus sequences corresponding mostly to clade 1a (which includes SARS-CoV) and 1b (which includes SARS-CoV-2) along with a clade 2 sequence [Rf1-CoV (GenBank DQ412042)]. There are ongoing studies for pan-betaCoV vaccines, yet details on the vaccine inserts are not available (*41*) (https://absolutelymaybe.plos.org/2022/07/09/front-runners-in-the-race-for-variant-proof-and-all-coronavirus-vaccines/#top). On the basis of publicly available data, it appears that the coverage target of a vaccine as described in press releases does not necessarily mean that the sequence insert comprises sequences from the corresponding genera. We are not aware of efforts toward a pan-CoV vaccine that would include strains encompassing diverse CoVs, and our results show that, from a genetic perspective, the space that needs to be covered is so vast and complex (because of recombination and differential selection) that a rational approach to do this seems difficult to develop. Our work follows a phylogenetic rationale to vaccine design, i.e., sequences from a given genera should be included if coverage of that genera is sought. While it is possible that a given vaccine platform would elicit immunity that would go beyond the genetic space covered by the inserts, our goal was to develop a genetic approach to identify the insert sequence(s) adapted to specific coverage of CoV strains irrespective of the potency of the responses elicited by a given platform. Further studies will be needed to characterize the immunologic space that each sequence covers and whether a combination of sequences allows immunologic gains in the antigenic map of CoVs.

## MATERIALS AND METHODS

### Sequences

Whole-genome nucleotide and S, E, M, and N amino acid reference sequences were downloaded from GenBank for seven hCoVs: SARS-CoV-2, SARS-CoV, MERS-CoV, HKU1, OC43, NL63, and 229E. The Virus Pathogen Database and Analysis Resource (www.viprbrc.org) was used to download genomic nucleotide sequences for 2181 nonhuman-infecting Coronaviridae strains and amino acid sequences for S (*n* = 1247), E (1235), M (1241), and N (1245). Sequences were removed if they were duplicates (>99% identity) or incomplete.

### Alignments

Sequences were aligned using Multiple Alignment using Fast Fourier Transform (MAFFT) v7.475 (*42*), first by genera, and then genera were aligned using --add. Subunit coordinates were obtained from GenBank for betaCoVs and alphaCoV and separately for 229E (*43*) and NL63 (*44*). The S1, RBD, and S2 alignments were made by aligning nonhuman S sequences to each hCoV separately with the --keeplength parameter in MAFFT v7.475.

### Phylogenetic reconstruction

A phylogeny was constructed for the whole-genome nucleotide alignment using FastTree 2 with a generalized time-reversible model (*45*). For protein phylogenies, recombination breakpoints were identified using genetic algorithm for recombination detection (*46*), which searches for phylogenetic incongruence among partitions of the alignment. Genera were analyzed separately for S. For E, M, and N, which are short in length (<600 amino acids), alignments were iteratively downsampled 100 times to the maximum allowable sequences for recombination analysis (20, 49, and 103, respectively); breakpoint positions were averaged across iterations. Phylogenies were then constructed using IQ-TREE 2 (*47*) with best-fit models inferred using ModelFinder (*48*) and data partitioned according to the inferred recombination breakpoints. A dengue virus 1 polyprotein (QNL13511.1) was included as an outgroup to root phylogenies.

### Missing diversity estimation

In ecology, species richness is estimated from the number of species sampled within an assemblage. By counting the number of individuals of each identified species in an assemblage, the number of missing species can be estimated on the basis of the abundance distribution of individuals sampled (*49*). The important factors for estimating richness are the unit being counted (e.g., species), the limits of the sample (e.g., species assemblage), and the shape of the abundance distribution curve (*50*). Viruses, which have a population structure that is fundamentally distinct from either species in a forest or microbial operational taxonomic units, cannot be counted as individuals belonging to species. We therefore designed an approach, adapted from a nonparametric method (*49*), to estimate the missing residues (unit) at each site (assemblage) in a viral alignment assuming a truncated log-normal distribution of residues at each site (distribution curve). The aim of this approach was to estimate the expected diversity of amino acids at each site in a protein alignment. For a given site in the alignment, the distribution of amino acids was assumed to follow a truncated log-normal distribution because the maximal richness (i.e., possible number of different amino acids) is known (*51*). The estimated diversity of amino acids, *D*_e_, is then estimated at each site as

$$D=D_{o}+\frac{f_{1}^{2}}{2f_{2}}$$

$$D_{\mathrm{var}}=f_{2}[\frac{1}{2}{(\frac{f_{1}}{f_{2}})}^{2}+(\frac{f_{1}}{f_{3}})+{(\frac{f_{1}}{f_{2}})}^{4}]$$

$$\sigma^{2}=ln\left[1+\frac{D_{\mathrm{var}}}{{\left(D-D_{o}\right)}^{2}}\right]$$

$$\text{Φ}=\int_{-\infty }^{x}f_{x}(t)dt$$

$$f(x|\mu ,\sigma )={\left(\sigma \sqrt{2\pi }\right)}^{-1}e^{\left[\frac{-{\left(x-\mu \right)}^{2}}{2\sigma^{2}}\right]}$$

$$\Gamma =\Phi \left[\frac{ln(D_{\mathrm{ul}}-D_{o})-ln(D-D_{o})}{\sigma }\right]$$

$$D_{e}=D_{o}+(D-D_{o})e^{\left\{\frac{\sigma }{\Phi }[\Gamma \left(\frac{\alpha }{2}\right)]\right\}}$$

where *f*_1_ is the number of minimally occurring amino acids, *f*_2_ is the number of the next minimally occurring amino acids, *D*_o_ is the number of observed amino acids, *D*_var_ is the variance of *D*_o_, and *D*_ul_ is the number of possible amino acids (upper limit).

#### Simulated alignments for testing missing diversity estimation

Alignments were simulated under a K80 model with between 5000 and 50,000 sequences, transition rates between 0.01 and 0.51, and transition/transversion rates of 0.5. Fifty alignments were simulated under 60 combinations of the sequence number and rate parameters. Alignments were randomly downsampled for sampling fractions =1 − 91%. *D*_e_ was then recovered using the above equation, and mean differences of simulated and estimated amino acids at each site were compared for each simulated scenario.

#### Missing amino acid diversity in protein alignments

The number of estimated missing amino acids (*D*_e_ − *D*_o_) at each site in the E, M, and N protein alignments was computed, as well as in S for each genus separately. The most likely missing amino acids at each site (if estimated to be any) were imputed using empirical transition rate matrices calculated for each protein (and for S, genus).

### Sequence and phylogenetic analysis

Pairwise amino acid distances between sequences were determined using dist.ml (*52*) with a JTT substitution model. The correlation coefficient between phylogenies was estimated using the Goodman-Kruskal γ index (*53*) and 50 permutation tests in the R package dendextend v1.15.1 (*54*) between protein phylogenies and between each protein phylogeny and the whole-genome phylogeny after removing any strains not shared between phylogenies. Molecular rates were estimated using uncorrelated clocks and bootstrapped confidence intervals (*55*) for protein phylogenies and genus-specific protein phylogenies; pairwise comparisons between rates were done using pairwise *t* tests with a Bonferroni correction.

### Ancestral sequence reconstruction

Ancestral sequences were reconstructed at all internal nodes using FastML v.3.11 with branch-length optimization and a gamma distribution (*56*) for each protein alignment. Differences between contemporary sequences and the sequence of the MRCA were determined using the amino acid reconstructions with the highest marginal probability at each site.

### Identifying RBD sequence similarity that diverges from a null expectation

We developed a method for scoring sequence similarities (i.e., sequence identity scores) between RBDs that weighted phylogenetic distance between hCoV RBDs and each wild strain RBD. This method leveraged the evolutionary dynamic observed in S for betaCoVs, which is that sequence conservation is twice as high in S2 than the RBD.

The coordinates for the RBD and S2 segment of the S protein were retrieved from GenBank for hCoVs. The RBDs and S2s of human-infecting betaCoVs were aligned separately, and then nonhuman-infecting betaCoV sequences were aligned to them using the keeplength option in MAFFT v7.475 (*42*). A phylogeny was constructed from the S2 alignment using IQ-TREE 2 (fig. S6A) (*47*). Sequences were simulated 1000 times along the S2 phylogeny using a continuous-time Markov process (*57*), seeding the simulation with the ancestral reconstruction of the RBD (fig. S6B), and then computing the distance matrices for the simulated sequences (fig. S6C). This was done to simulate RBD-like sequences assuming the more neutral evolutionary history of S2. A phylogeny was constructed for the RBD alignment as above (fig. S6D), and a distance matrix was computed for the RBD sequences (fig. S6, D and E).

For each hCoV, the actual genetic distances to wild strain RBDs were regressed against distances in the simulated alignments, forcing the intercept to zero (fig. S6F). The negatives of the residuals of the regression were calculated for each wild strain (fig. S6G). Negatives were taken so that a higher residual indicated an RBD distance smaller than expected given the simulated distance between a human and wild sequence. Residual scores were averaged over 1000 simulations for each hCoV.

Site-specific amino acid conservation was calculated between the human-infecting sequence and nonhuman-infecting sequences for each hCoV. RBD sites of interest were defined as sites more conserved in sequences in the second quartile for residual scores of >0 (+*Q*_2_) compared to sequences in the second quartile for residual scores of <0 (−*Q*_2_).

### Extrapolating sequences for a pan-betaCoV vaccine design

Strains in the top 5% of RBD residual scores for each hCoV (so-called human-like RBDs) were aligned to hCoV RBDs. A phylogenetic tree was constructed using IQ-TREE 2 (*47*) with best-fit models inferred using ModelFinder (*48*). After removing hCoV sequences, the distance matrix of the alignment was clustered on k-medoids using optimal silhouette width, *s*(*i*), which is a measure of the between/within variance of each datapoint *i* assigned to a cluster; data are typically considered to have a discretized structure, defined by excessive variance between groups and minimal variance within groups, at $\bar{s}>0.51$(*58*, *59*). For each cluster of sequences, a majority consensus sequence was inferred; when no majority residue was present, the most abundant residue that was different from the nearest-neighbor hCoV(s) was taken; all inferred gaps were removed.

### Structure prediction of cluster sequences

To predict the protein structure of derived cluster consensus sequences, prediction alignment error and local distance difference tests were computed for each sequence in ColabFold (*60*), which uses AlphaFold2-ptm for structure prediction and AlphaFold-multimer for complex prediction (*61*). Each sequence was aligned to UniRef100 and environmental sequences with MMseqs2 (*62*).

## Notes

View/request a protocol for this paper from *Bio-protocol*.
