# Supplementary material for: Design of a pan-betacoronavirus vaccine candidate through a phylogenetically informed approach
Source: Sci Adv. 2023 Jan 18;9(3):eabq4149. doi: 10.1126/sciadv.abq4149 (PMC9848278; doi:10.1126/sciadv.abq4149)
Supplement: Supplementary file 1 — Table S1 Figs. S1 to S8 [file sciadv.abq4149_sm.pdf]

Science Advances
AAAS

Supplementary Materials for

Design of a pan-betacoronavirus vaccine candidate through a phylogenetically informed approach

Eric Lewitus *et al.*

Corresponding author: Morgane Rolland, mrolland@hivresearch.org

Sci. Adv. **9**, eabq4149 (2023)
DOI: 10.1126/sciadv.abq4149

The PDF file includes:

Table S1
Figs. S1 to S8
Legends for files S1 and S2

Other Supplementary Material for this manuscript includes the following:

Files S1 and S2

Supplementary Materials

hCoV	No. strains with residual $\geq Q_2$	No. strains with residual $\leq -Q_2$	No. sites of interest
SARS-CoV-2	51	42	57
SARS-CoV	37	43	113
MERS-CoV	29	65	23
HKU1	29	66	40
OC43	33	60	25

Table S1: Number of strains in the top 25th ($+Q_2$) and bottom 75th ($-Q_2$) percentile of RBD residual scores and number of sites more conserved in the 2nd quartile of $+Q_2$ than the 2nd quartile of $-Q_2$ for each hCoV.

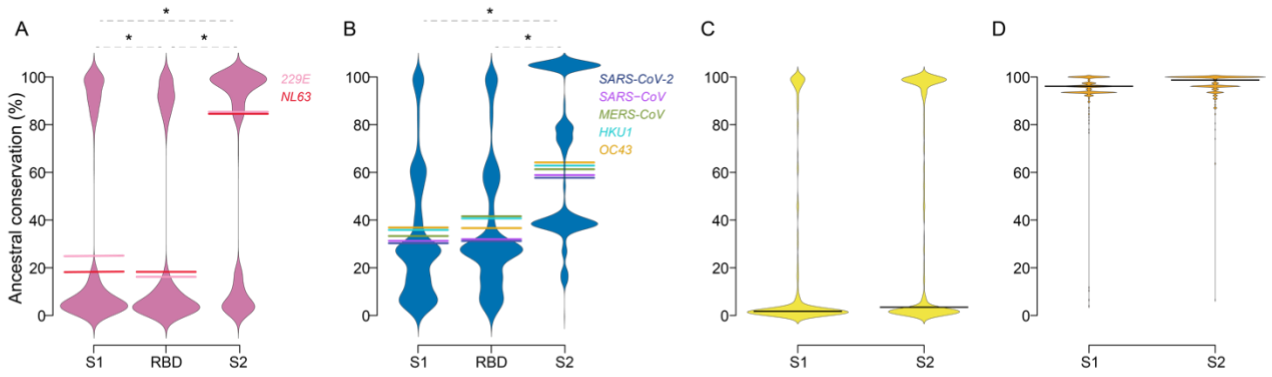

Figure S1: Ancestral conservation within S across genera. Violin plots of the percentage of ancestral site conservation across (A) alphaCoV, (B) betaCoV, (C) gammaCoV, and (D) deltaCoV. The percentage conservation for hCoVs is shown in (A,B). Asterisks indicate pairwise significant differences (Mann-Whitney U test, P<0.05).

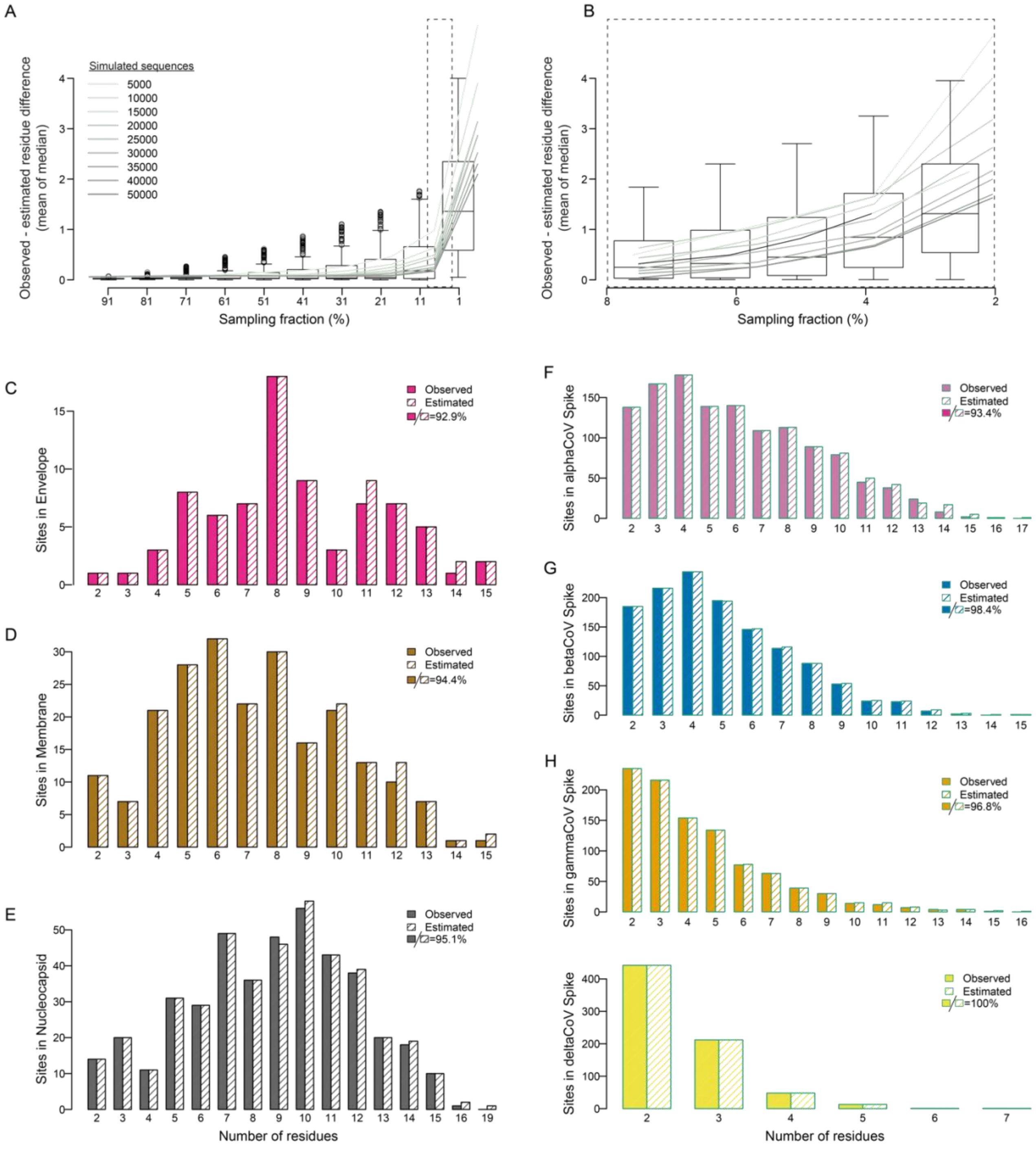

Figure S2: Large yet representatively sampled diversity across hCoVs. (A) Boxplots of mean of median difference estimates (D_o-D_e) across simulated alignments by sampling fraction. Lines indicate median values for alignments of different size (number of sequences, see Legend). (B) Resolution on sampling fractions < 10%. Means were computed on median values within alignments of each size. (C-I) Barplot of the number of sites with different numbers of observed (D_o, filled) and estimated (D_e, striped) residues for alignments of (C) Envelope, (D) Membrane, and Nucleocapsid (E) sequences for all coronavirus genera; and for alignments of (F) alphaCoV, (G) betaCoV, (H) gammaCoV, and (I) deltaCoV Spike sequences. The median observed diversity as a percentage of the estimated diversity across sites is shown for each barplot.

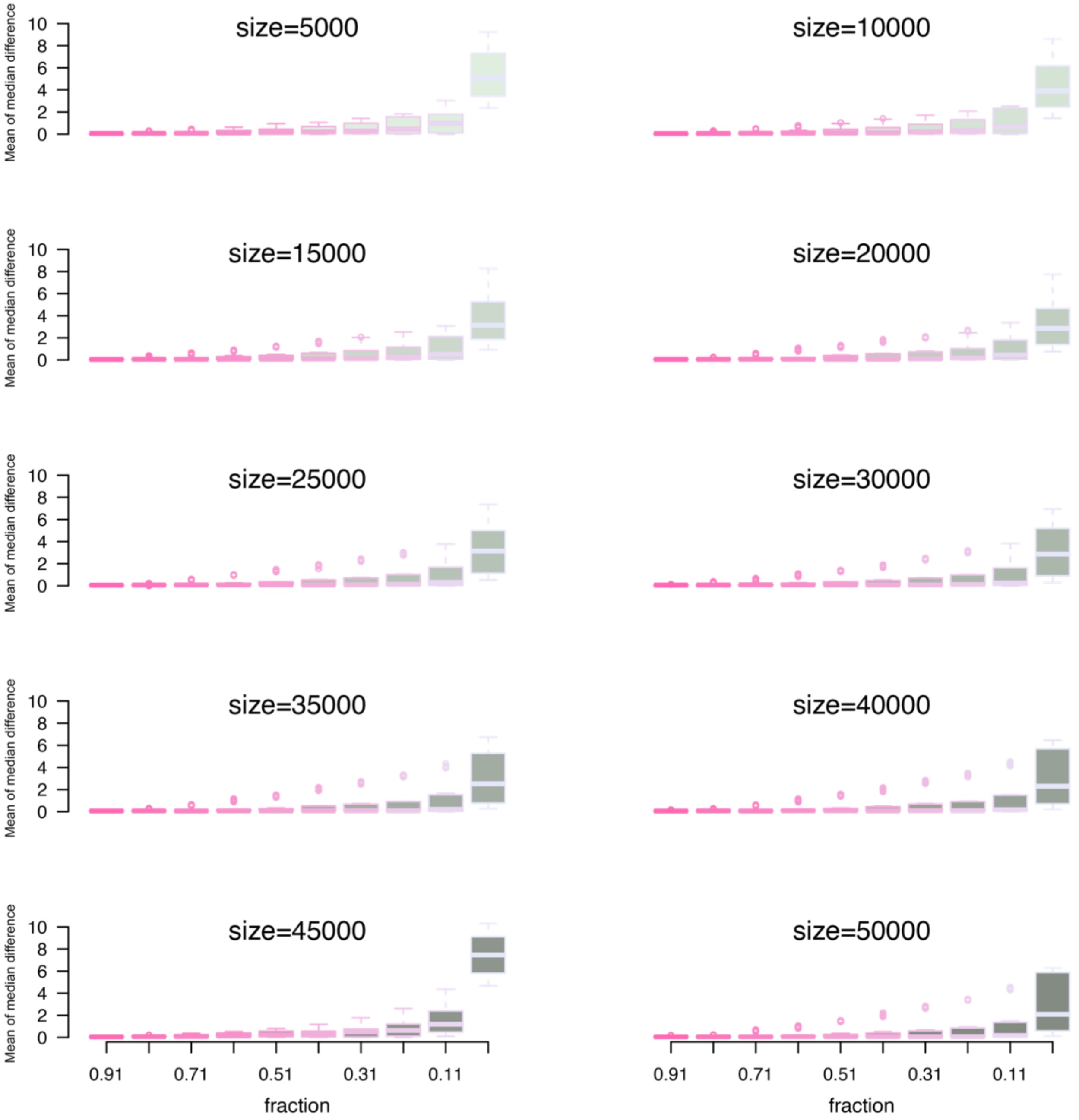

Figure S3: Diversity estimates in different sizes of simulated alignments. Boxplots of mean of median difference estimates (D_o - D_e) across simulated alignments of different sizes by sampling fraction. Means were computed on median values within alignments of each size.

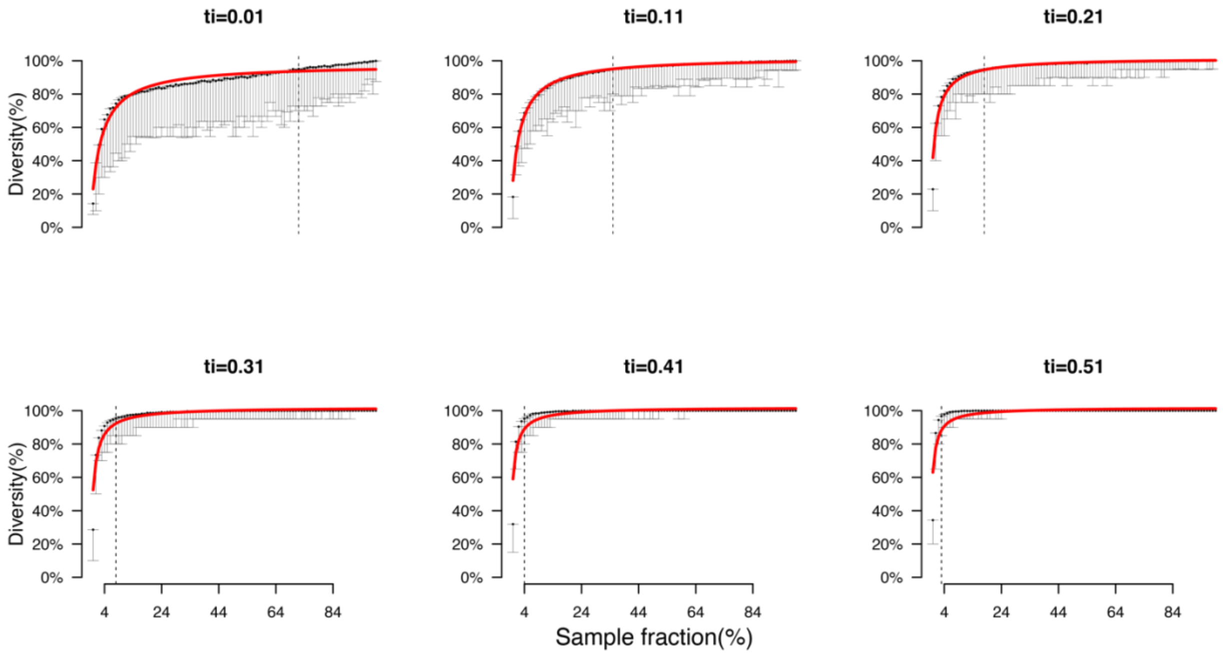

Figure S4: Rarefaction curves for alignments simulated under different transition rates. Dashed lines indicate the sampling fraction needed to recover 90% diversity from 100 samples.

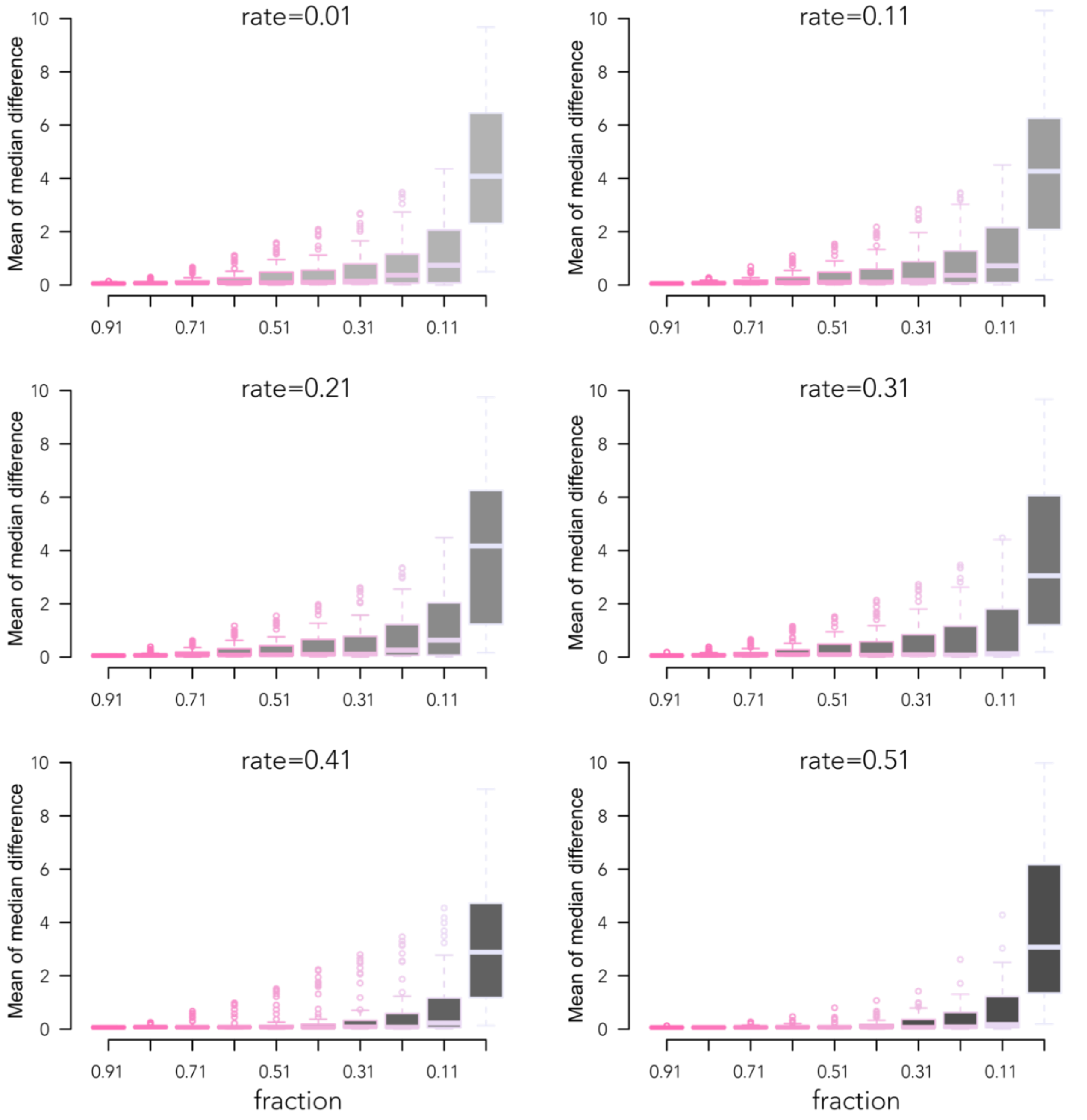

Figure S5: Diversity estimates in alignments simulated under different transition rates. Boxplots of mean of median difference estimates (D_o-D_e) across alignments simulated under different transition rates by sampling fraction. Means were computed on median values within alignments of each transition rate.

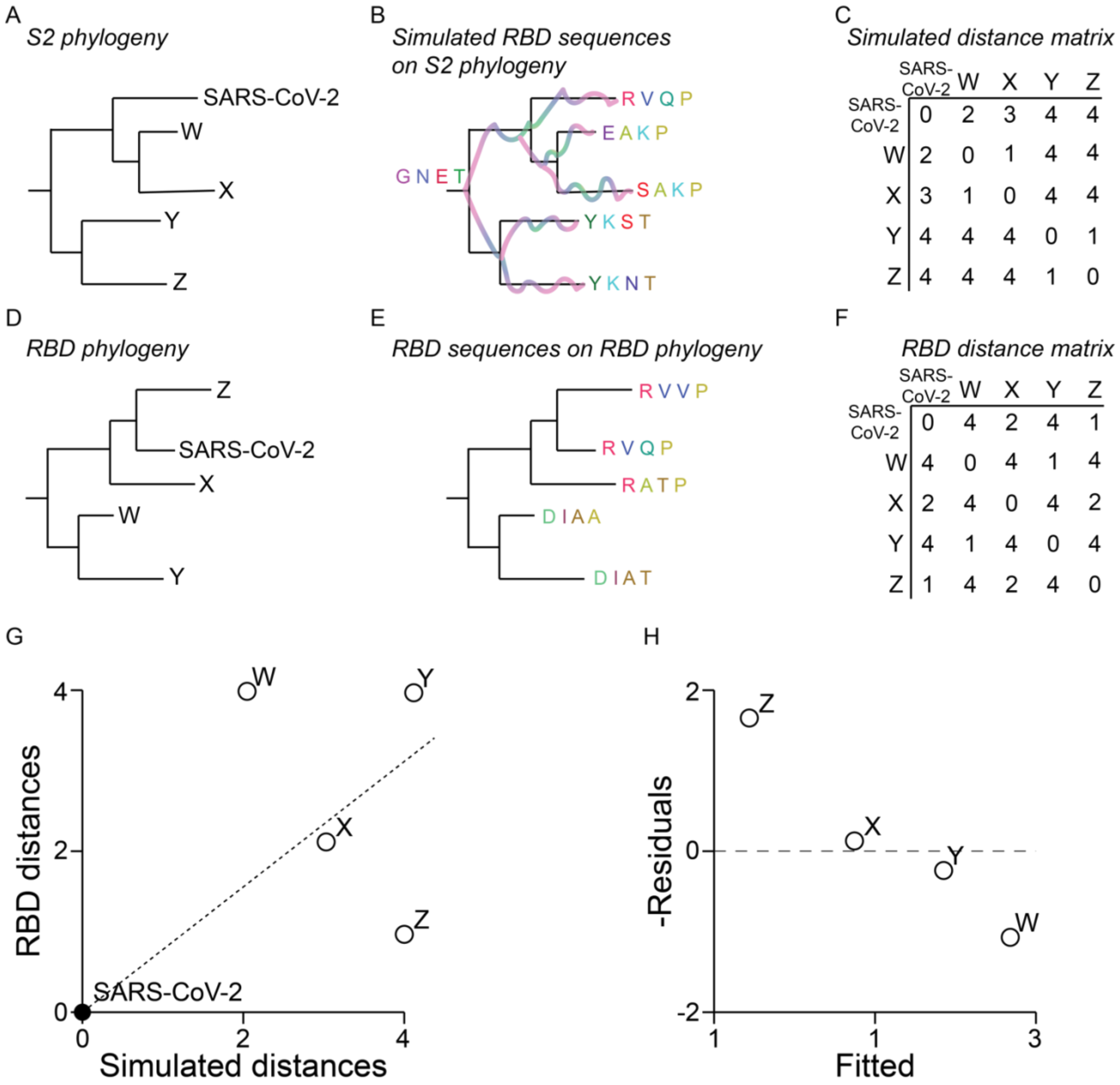

Figure S6: Schematic for computing RBD residual scores. (A) A phylogeny constructed based on S2 sequences, (B) sequences evolved on the S2 phylogeny seeded with the RBD MRCA, and (C) a distance matrix calculated on the simulated RBD sequences. (D) A phylogeny constructed from RBD sequences, (E) RBD sequences represented on the RBD phylogeny, and (F) a distance matrix calculated on RBD sequences. (G) Plot of RBD distances from the hCoV of interest (e.g., SARS-CoV-2) as a function of simulated RBD distances from the same hCoV; the dashed line shows the least-squares regression with a forced intercept at zero. (H) Negative of the residual scores computed from the regression in (G), where higher residual scores indicate that the distance in (G) along the y-axis (i.e., the actual distance between the hCoV RBD and the wild strain RBD) is smaller than expected given the distance along the x-axis (i.e., the distance between the hCoV RBD and simulated RBD for the wild strain).

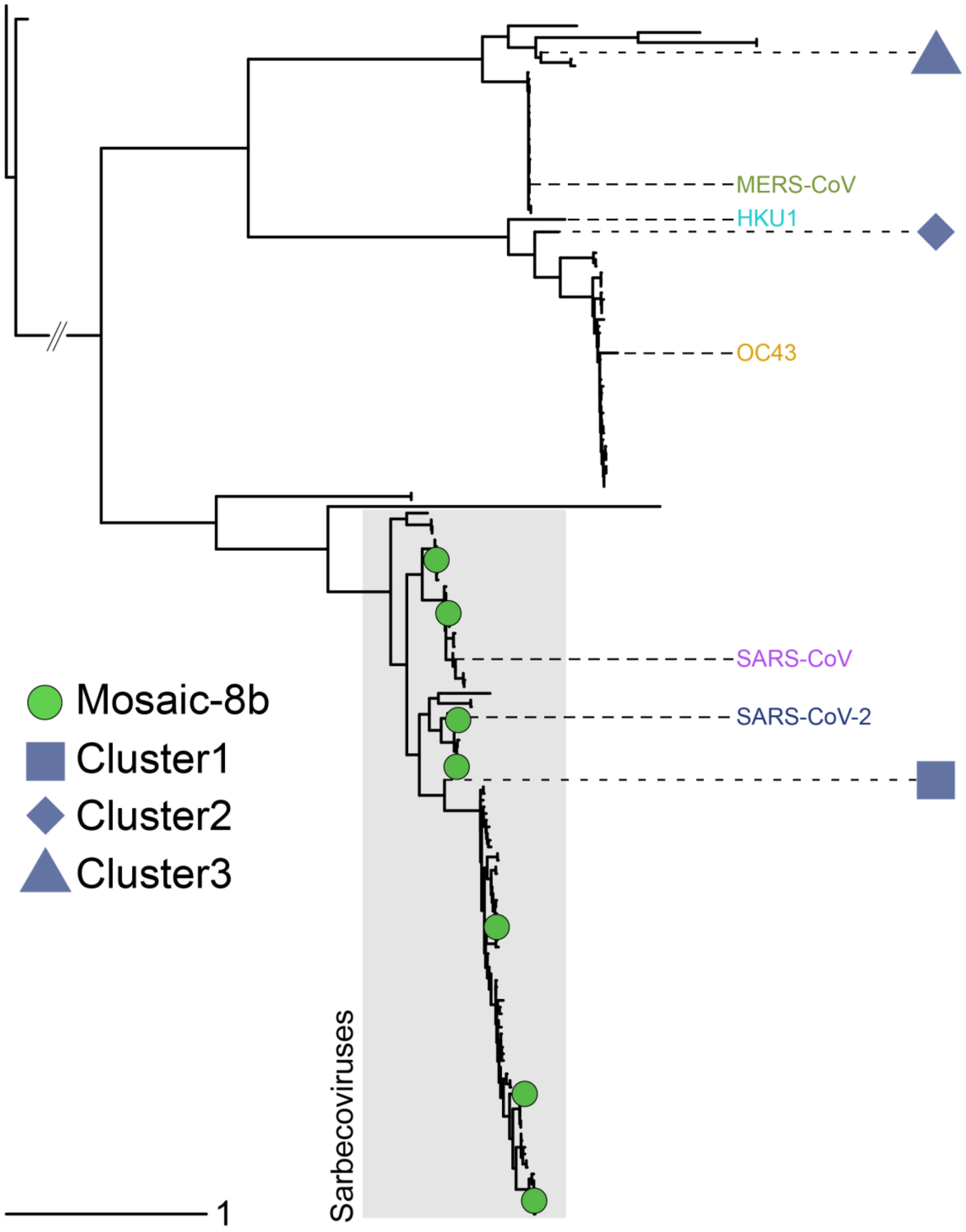

Figure S7: **Phylogeny of 185 betaCoV RBD protein sequences**. These include human-infecting sequences, cluster consensus sequences (blue) and eight non-human-infecting sequences included in the Mosaic-8b vaccine developed by Cohen and colleagues *(29)* (green). Sarbecoviruses are highlighted in grey. The tree is rooted on the subgenus Nobecovirus (YP_009273005.1), the phylogeny scale bar is shown.

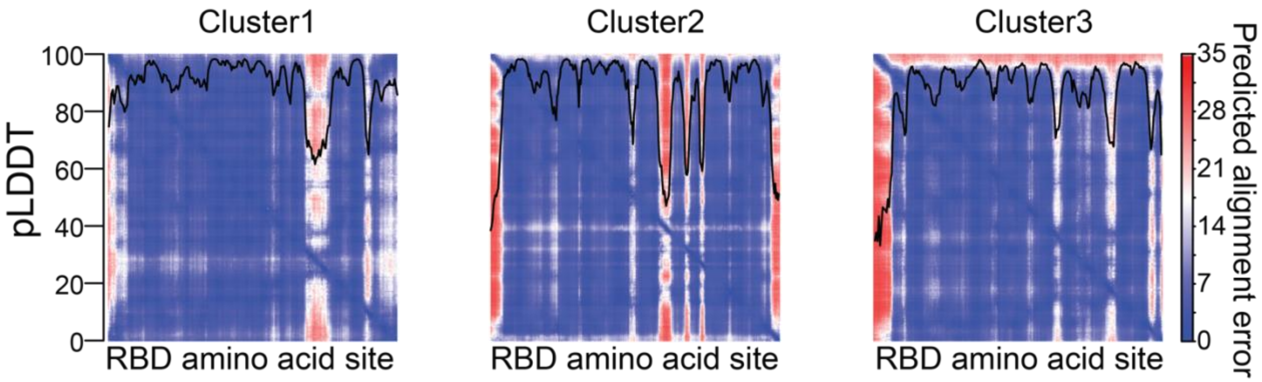

Figure S8: **Heatmaps of predicted alignment error and line-plots of predicted local distance difference tests (pLDDTs) for all sites in derived cluster consensus sequences.**

Supplemental File Legends

File S1: Clustal alignment of betaCoV hCoVs and human-like RBDs.

File S2: Human-like RBDs cluster consensus sequences.
